# Design Requirements for Gamified Exercise Apps for Adults With Prehypertension Based on the Octalysis Framework and Self-Determination Theory: Qualitative Interview Study

**DOI:** 10.2196/86793

**Published:** 2026-02-25

**Authors:** Sijia Li, Genying Liu, Li Qiao, Hong-Yu Yu, Yu-Yang Zhang, Runyuan Pei, Yue-Tao Lin, Hao-Ming Ma, Mei-Hua Piao

**Affiliations:** 1 School of Nursing, Chinese Academy of Medical Sciences & Peking Union Medical College, 33 Badachu Road, Shijingshan District, Beijing, 100144, China, 86 13522112889; 2 Outpatient Department, West China Hospital, Sichuan University, Chengdu, China; 3 School of Nursing, University of Washington, Seattle, WA, United States; 4 Comprehensive Disease Center, West China Xiamen Hospital of Sichuan University, Fujian, China

**Keywords:** prehypertension, gamification, mHealth, qualitative study, self-determination theory, mobile health

## Abstract

**Background:**

Managing blood pressure (BP) in prehypertensive individuals is crucial to prevent the incidence of hypertension. While physical activity has proven effective in BP management, physical inactivity remains prevalent. Gamification has shown promise in addressing physical inactivity; however, its effectiveness is limited due to the suboptimal intervention design.

**Objective:**

This study aimed to develop a comprehensive understanding of prehypertensive individuals’ needs, design preferences, and motivational drives for gamified exercise apps by integrating the Octalysis Gamification Framework with Self-Determination Theory (SDT) through a participatory qualitative methodology.

**Methods:**

The study adopted a cross-sectional qualitative interview study design and used semistructured interviews conducted from June to July 2025 across mainland China. Participants were recruited through online platforms using purposive sampling to select adults with prehypertension (systolic BP 120‐139 mm Hg and/or diastolic BP 80‐89 mm Hg). Interview guides were systematically structured around SDT constructs and the Octalysis Framework’s 8 core gamification drives to explore participants’ needs, design preferences, and motivational drives for gamified exercise apps. An inductive or deductive hybrid thematic analysis was used to identify key themes.

**Results:**

A total of 14 eligible prehypertensive individuals participated in the study. Their needs and preferences for exercise apps, including exercise guidance, data monitoring and feedback, and wearable device portability, were summarized. Eight core gamification motivational drives, such as epic meaning and social influence, according to the Octalysis Framework, were explored with interviewees, including their attitudes and creative design considerations. Moreover, the study examined how the satisfaction of basic psychological needs (eg, sense of volition, technical challenges, and social connections) influences the transformation of motivation.

**Conclusions:**

This theory-informed qualitative study is among the first to explore the needs and preferences of individuals with prehypertension for gamified exercise apps by integrating the Octalysis Gamification Framework with SDT. The findings suggest that successful gamified exercise apps for prehypertensive individuals may benefit from going beyond traditional game mechanics to address deeper psychological needs. Participants emphasized the importance of personalized exercise programming, robust health monitoring capabilities, and human-centered gamification designs that support autonomy, competence, and relatedness. Satisfaction of these basic psychological needs is critical for transforming extrinsic motivations, such as weight management and health values, into intrinsic enjoyment of exercise. By integrating a theoretical basis with user-centered perspectives, this study provides context-specific design implications for future development of gamified exercise apps tailored to prehypertensive individuals in similar digital health contexts.

## Introduction

Hypertension is the leading risk factor for global disease burden [1]. The World Health Organization (WHO) estimated that 1.28 billion adults aged 30‐79 years are affected by hypertension worldwide. Additionally, the WHO aims to reduce the prevalence of hypertension by 33% from 2010 to 2030, a goal that necessitates effective hypertension prevention strategies [2]. Data from a randomized controlled trial indicate that two-thirds of untreated prehypertension cases can progress to hypertension within 4 years [3]. Managing blood pressure (BP) in prehypertensive individuals is crucial to prevent the incidence of hypertension. Lifestyle interventions, such as adopting physical activity (PA) programs, are strongly recommended in clinical guidelines for managing BP among individuals with prehypertension [4]. In addition, a previous clinical trial involving prehypertensive individuals found that Tai Chi reduced systolic BP by 7.01 mm Hg and diastolic BP by 3.73 mm Hg, demonstrating notable effectiveness [5]. Although PA has shown effectiveness in BP management in both clinical guidelines and randomized controlled trials, physical inactivity remains prevalent, with one-third of adults being insufficiently physically active in 2022 [6]. This disconnect between evidence and practice presents a significant challenge in cardiovascular disease prevention.

Digital health technologies offer scalable solutions to this challenge. Gamification—the strategic integration of game elements such as points, levels, badges, and avatars into nongame contexts such as health care, education, and training—has shown promise in addressing physical inactivity [7]. The meta-analysis demonstrated a small to medium effect (Hedges *g*=0.42) of gamification on PA, though notably, long-term effects diminish significantly, and current interventions have not translated into measurable health benefits [8,9]. This limited effectiveness stems from 3 critical gaps: interventions often lack robust theoretical foundations, fail to incorporate user-centered design principles, and neglect the psychological mechanisms underlying sustained behavior change [10,11]. Therefore, given the current limitations of gamification intervention design and its limited effectiveness, our team is developing a gamified exercise app that is theory-driven, evidence-based, and incorporates user perspectives. This qualitative study, based on user interviews, is part of the broader research topic. This interview aimed to understand the needs, design preferences, and motivations of prehypertensive individuals for gamified exercise apps.

In contrast to prior approaches that rely on either gamification frameworks or psychological theories in isolation, we integrated both to provide a deeper understanding of how gamification elements relate to users’ psychological need satisfaction and support health behavior change [12,13].

First, we selected the Octalysis Gamification Framework as our primary theoretical basis for analyzing gamification mechanics. Created by Chou in 2019 [14], this human-focused framework is well known for emphasizing motivational drives rather than functional features, identifying 8 core drives that underlie user motivation in gamified systems. However, while Octalysis explains what motivates users, it provides limited insight into how these motivations translate into sustained behavioral engagement.

To address this, we integrated Self-Determination Theory (SDT), a well-established motivational theory, to elucidate the psychological pathways through which external gamification elements may foster intrinsic motivation [15]. SDT offers a theoretical lens for linking gamification drives to the satisfaction of 3 fundamental psychological needs—autonomy, competence, and relatedness—with specific Octalysis core drives (eg, achievement and social influence) mapping to distinct needs (eg, achievement to competence), thereby facilitating the transition from extrinsic to intrinsic motivation.

By combining Octalysis and SDT, this dual-theory approach enables us to bridge the gap between surface-level gamification features and deep psychological engagement [16]. Guided by this, we conducted a user-centered qualitative study to understand the needs of prehypertensive individuals in relation to exercise apps, as well as to explore gamification design considerations.

Specifically, our objectives were to (1) explore the needs and design preferences for exercise apps among individuals with prehypertension, (2) identify optimal gamification design strategies based on the Octalysis Framework from users’ perspectives, and (3) elucidate how exercise apps can support motivational transformation in the target population according to SDT. The insights derived from this interview will inform the development of next-generation gamified health interventions and contribute to the broader discourse on theory-informed digital health design.

## Methods

### Overview

This study used a cross-sectional qualitative interview study design grounded in a pragmatic and interpretive orientation, using semistructured interviews to generate user-centered insights. This approach was chosen to generate practical design implications for gamified exercise apps while facilitating an interpretive understanding of participants’ exercise-related experiences. To ensure adherence to qualitative research reporting standards, we followed the COREQ (Consolidated Criteria for Reporting Qualitative Research) checklist (Checklist 1) and the American Psychological Association Journal Article Reporting Standards for Qualitative Research (JARS-Qual) [17,18].

### Study Setting and Recruitment

The study was conducted from June to July 2025 through online poster recruitment in mainland China. To ensure the richness and relevance of the findings, purposive sampling was used to select individuals with prehypertension [19]. We aimed to achieve gender balance and include individuals from diverse socioeconomic backgrounds and occupations in our recruitment. The sample size was determined in accordance with the principle of data saturation. Interviews were discontinued after a minimum of 10 sessions, when no new codes or themes emerged after 3 consecutive interviews [20].

Inclusion criteria included the following: (1) aged between 18 and 60 years old; (2) met the 2024 Chinese diagnostic criteria for prehypertension, defined as systolic BP between 120 and 139 mm Hg and/or diastolic BP between 80 and 89 mm Hg [21]; (3) able to communicate verbally in Mandarin and willing to participate voluntarily; (4) expressed an interest in the association between exercise and BP; and (5) had prior experience using smartphone apps. Exclusion criteria were as follows: (1) individuals with any known cognitive impairments or mental illnesses that could affect their ability to participate in an interview and (2) individuals with physical conditions that may impact their PA behavior.

### Theoretical Framework

The interview guidelines were systematically developed through integration of SDT and the Octalysis Gamification Framework, encompassing questions regarding 3 aspects: exercise behavior, needs, and preferences for exercise apps, and gamified incentive mechanisms (Multimedia Appendix 1) [14,16]. According to SDT, human motivation undergoes a continuous transition from amotivation to extrinsic motivation and then to intrinsic motivation. When the external environment provides appropriate support and satisfies an individual’s basic psychological needs (autonomy, competency, and relatedness), it can stimulate intrinsic motivation or facilitate the transformation of extrinsic motivation [16,22]. The Octalysis Gamification Framework, developed by Chou in 2019, has been widely adopted across diverse fields and is recognized for emphasizing human motivation rather than mere functionality to improve the impact of gamification [14,23]. It categorizes game elements into 8 fundamental psychological drives (ie, epic meaning, accomplishment, empowerment, ownership, social influence, scarcity, unpredictability, and avoidance) that shape human behavior and decision-making. These core drives serve to motivate, empower, and influence behavioral patterns, ultimately prompting individuals to take action.

**Figure 1.**
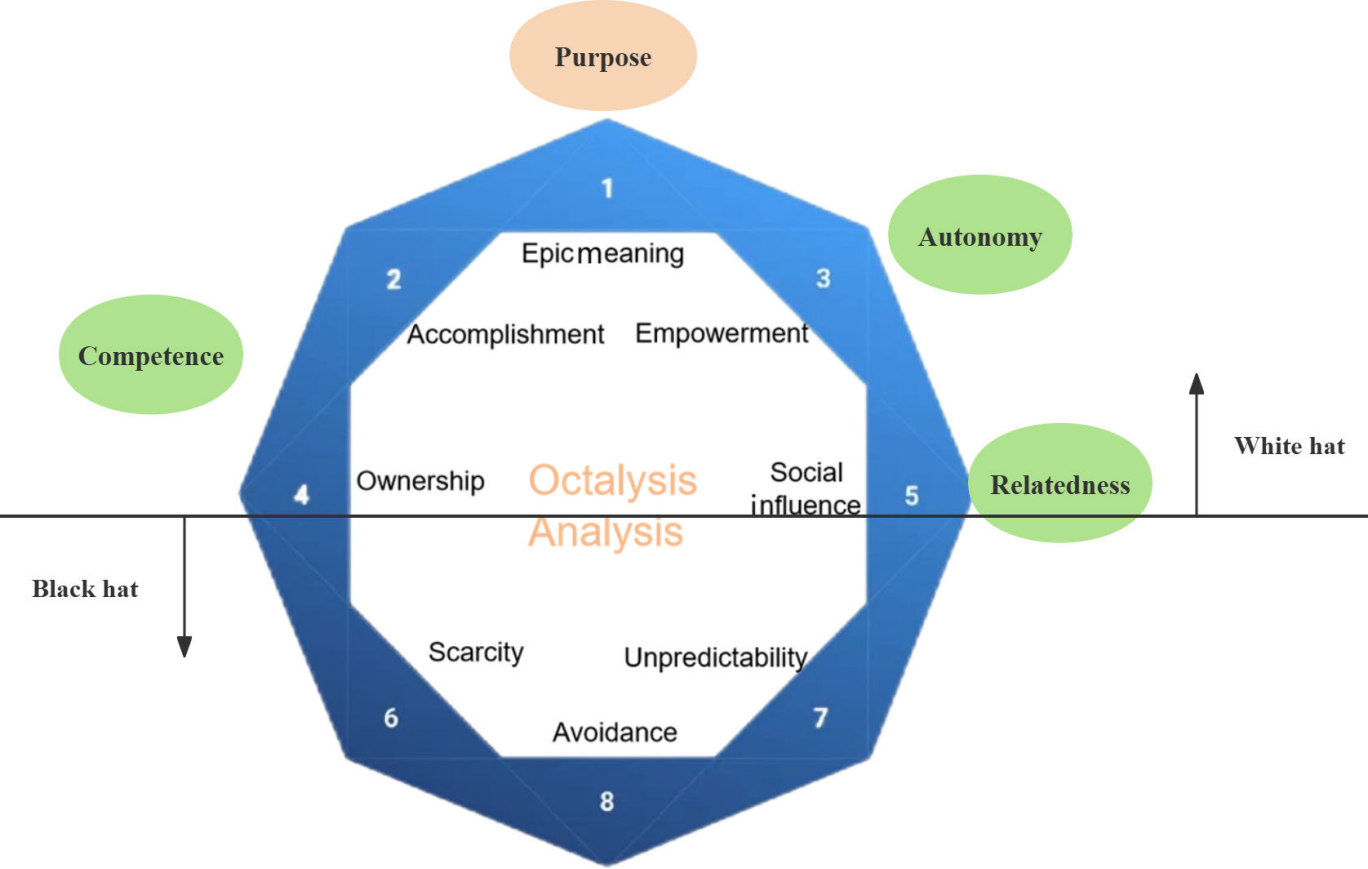
Octalysis and Self-Determination Theory relationship.

The relationship between the Octalysis Framework and SDT is illustrated in Figure 1. The figure conceptually depicts how selected white hat Octalysis motivational drives may be interpreted through an SDT-informed lens in relation to basic psychological needs of autonomy (empowerment), competence (accomplishment and ownership), and relatedness (social influence), as well as a sense of purpose (epic meaning). Ownership is depicted as having a weaker association with competence, as it often relies on responsibility rather than intrinsic mastery. The horizontal axis distinguishes between predominantly psychological need support (white hat) and more externally driven (black hat) motivation. Black hat drives (scarcity, avoidance, and unpredictability) are shown as operating outside psychological need satisfaction and are therefore not theoretically aligned with SDT.

This dual-theory approach enabled us to systematically explore both the functional features of gamification (Octalysis) and its psychological aspects (SDT) in health behavior change.

### Data Collection

Prior to data collection, the interview guide was rigorously reviewed by 3 qualitative methodology experts, pilot-tested with 2 prehypertensive individuals, and iteratively finalized. Data were gathered through one-on-one semistructured interviews, which were conducted by a trained qualitative researcher (SJL), a female master’s student with 2 years of experience in health behavior research and a research interest in gamified exercise apps, to ensure consistency. The researcher’s prior understanding of behavior change theories and gamification informed the development of the interview guide. Interviews were conducted either through online Tencent Meetings (Tencent) or face-to-face in a quiet meeting room with no other person present, depending on the specific circumstances and participant preferences. Before the interviews, the researcher provided participants with an explanation of the topic, reasons, and purposes for doing the research, and privacy protections to foster trust with the participants. Participants were also invited to complete demographic questionnaires prior to the interview. Those participating in face-to-face interviews completed paper-based questionnaires, whereas those interviewed through Tencent Meetings completed questionnaires through Wenjuanxing (Changsha Ranxing Information Technology Co, Ltd), a widely used online survey platform in China.

During the interview, before discussing gamification, the interviewer introduced the concept to the participants using examples from well-known apps such as Starbucks (Starbucks Corporation) and Duolingo (Duolingo, Inc). Interviews took place in private, comfortable environments, with each session lasting approximately 30 to 60 minutes. After the interviews, the interviewer took notes on key information. With the participant’s consent, all sessions were audio-recorded and transcribed verbatim within 24 hours. The researchers then returned the transcriptions to the participants to verify the accuracy of the content.

### Data Analysis

The interviewer (SJL) transcribed the interview recordings verbatim using the transcription software iFlytek Hearing. Subsequently, the transcriptions were reviewed and verified for accuracy by listening to the audio recordings again in Microsoft Word, with any necessary corrections made based on the recordings. The finalized transcriptions were then uploaded to NVivo 20 (QSR International) for systematic qualitative data analysis, where coding and thematic analysis were conducted. Thematic analysis is a flexible method that can be applied to both inductive and deductive approaches to data analysis [24]. According to our research aim, we used an inductive or deductive hybrid thematic analysis, a methodologically rigorous approach that combines both theory-driven and data-driven approaches to identify key themes [25]. Specifically, inductive thematic analysis was used to explore the needs and preferences of prehypertensive individuals regarding exercise apps, while the deductive analysis was applied using predetermined codes derived from SDT and the Octalysis Gamification Framework. For example, SDT-informed deductive codes included autonomy (eg, sense of volition and preferences), while Octalysis-informed codes included motivational drives such as accomplishment (eg, points and progress bars).

Two researchers (SJL and HMM) with expertise in qualitative methods and health gamification independently coded the first 6 transcripts, then compared and discussed their results to establish preliminary themes and insights. In cases of disagreement, a third researcher (MHP) with extensive experience in behavioral health research was consulted to reach a consensus. The interviewer then proceeded to code the remaining transcripts, collaborating with the second researcher to incorporate any newly emerging themes. Finally, the entire research team reviewed and finalized the themes through iterative discussion until consensus was achieved.

### Methodological Rigor

Rigor was determined using Lincoln and Guba (1985) 4 criteria of credibility, dependability, confirmability, and transferability [26]. Credibility was established through the use of probing questions during interviews, such as “Can you elaborate?” These questions encouraged participants to provide more detailed responses, allowing researchers to gain a more comprehensive understanding of their experiences. Member checking, where participants were asked to review and confirm the accuracy of the interview data, was also used to ensure that the research findings reflected their true perspectives. An audit trail, which included a detailed record of how data were collected, kept, analyzed, and interpreted, was used to ensure dependability by providing a transparent research process and confirmability by ensuring that the qualitative findings were not influenced by researchers’ biases. Additionally, a detailed description of the research aim, participants, and context was provided to facilitate transferability to similar populations and settings.

### Methodological Integrity

Fidelity was ensured through consistent alignment among the research objectives, the guiding theoretical frameworks (SDT and the Octalysis Gamification Framework), the semistructured interview design, and the hybrid inductive-deductive thematic analysis. Utility was demonstrated by addressing a meaningful research problem and generating context-specific design implications to inform the future development of gamified exercise apps for individuals with prehypertension. These findings offer actionable insights for researchers and software designers working in digital behavior change contexts.

### Ethical Considerations

The study was approved by the Ethics Committee of Peking Union Medical College School of Nursing (approval number: PUMCSON-2024‐36) before data collection began and was conducted in compliance with the ethical principles outlined in the Declaration of Helsinki. Written informed consent was obtained from all participants after providing comprehensive information about study objectives, procedures, and data protection measures. All data were anonymized prior to analysis, and no information could be used to identify individual participants. Each interviewee received a compensation of 200 RMB (approximately US $28) as appreciation for their time. No identifiable images of participants were included in the manuscript or supplementary materials.

## Results

### Overview

The following results are based on interviews with 14 adults with prehypertension recruited online in mainland China.

#### Characteristics of Participants

A total of 14 individuals with prehypertension participated in the interviews. Seventeen individuals were invited in total, but 3 declined due to work commitments. We initially interviewed 11 participants, and after identifying the key topics, we conducted 3 additional interviews. Thematic saturation was reached, as no new themes or codes emerged in the final interviews. Data collection was therefore concluded. The characteristics of the 14 interviewed prehypertensive participants from mainland China are presented in Table 1. The mean age of participants was 33.93 (SD 12.86) years old, with the majority (64.29%) being female and single. The average BMI was 25.10 kg/m^2^ and the mean BP was 129 (SD 8)/81 (SD 6) mm Hg. All participants had at least a college degree and reported using the internet for approximately 8 hours daily. Nearly half of the participants engaged in regular exercise or frequently used their mobile phones.

**Table 1.** Characteristics of the prehypertensive participants from mainland China (N=14).

Variables	Value
Age (years), mean (SD)	33.93 (12.86)
Sex, n (%)	
Male	9 (64.29)
Female	5 (35.71)
BMI (kg/m^2^), mean (SD)	25.10 (4.28)
Marital status, n (%)	
Married	4 (28.57)
Single	9 (64.29)
Divorced	1 (7.14)
Educational background, n (%)	
College	11 (78.57)
Postgraduate	3 (21.43)
Occupation, n (%)	
Employee of an enterprise or institution	6 (42.86)
Student	4 (28.57)
Civil servant or official	1 (7.14)
Other	3 (21.43)
Daily internet use (hours), mean (SD)	8.64 (2.13)
Regular exercise habits, n (%)	
Yes	7 (50.00)
No	7 (50.00)
Mobile phone usage habits, n (%)	
Yes	6 (42.86)
No	8 (57.14)
Systolic or diastolic blood pressure (mm Hg), mean (SD)	129 (8)/81 (6)

#### Key Themes

##### Thematic Analysis Overview

**Figure 2.**
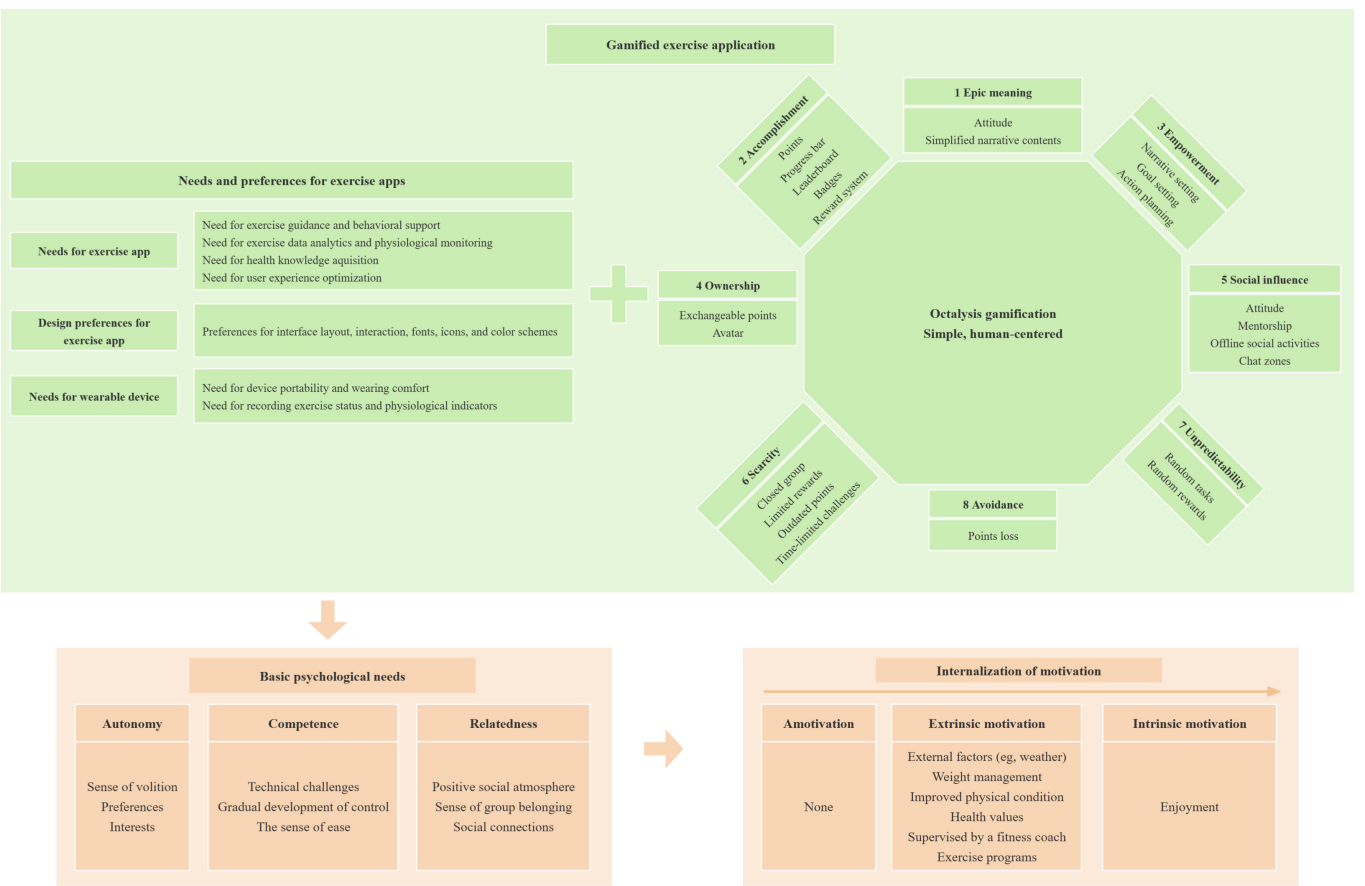
Conceptual framework in qualitative study.

Three overarching domains emerged from our hybrid inductive-deductive analysis, as shown in Figure 2. An inductive or deductive hybrid thematic analysis was used to analyze the data. Therefore, the theme of needs and preferences for exercise apps was divided into 3 subthemes: needs for exercise apps, design preferences for exercise apps, and needs for wearable devices. Based on the Gamification Octalysis Framework, 8 core psychological themes were predefined, with specific topics categorized under each core theme. The entire conceptual model was grounded in the SDT. The 3 fundamental psychological needs and motivation types were carefully identified and outlined.

##### Domain 1: Needs and Preferences for Exercise Apps

###### Overview

Themes, subthemes, and detailed quotations in domain 1 were summarized in Table S1 in Multimedia Appendix 2.

###### Needs for Exercise App

Participants identified several key needs for exercise apps, including real-time guidance, data feedback, and user-centered design. They expected the app to provide posture correction, integrate with calendars for automated reminders, and offer individualized exercise plans with clear progress schedules. Comprehensive tracking of exercise performance, calorie expenditure, and physiological indicators such as BP was also viewed as essential for maintaining motivation and monitoring health outcomes. In addition, participants emphasized intuitive onboarding, practical and content-oriented interfaces, and personalized interaction features that customized to different user preferences to enhance overall usability.

###### Design Preferences for Exercise App

Participants suggested that exercise apps should feature a more user-friendly and visually appealing interface, with simple fonts, intuitive icons (eg, a running figure), and color schemes such as green to evoke nature or orange to convey energy and vitality. They also emphasized the value of integrating conversational features powered by large language models to enhance personalization and sustain user engagement.

###### Needs for Wearable Device

Participants emphasized that wearable devices should be lightweight, comfortable, and focused on essential functions. They valued features such as exercise tracking, physiological monitoring, and alert notifications for indicators such as heart rate and BP, noting that these functions enhance safety and promote consistent exercise engagement.

### Domain 2: Gamification Design Insights Based on the Octalysis Framework

#### Overview

Themes, subthemes, and detailed quotations in domain 2 are shown in Table 2. Participants generally expressed positive attitudes toward incorporating gamified elements into exercise apps but emphasized that such features should remain simple, time-efficient, and seamlessly integrated into daily life to maintain practicality.

According to the Octalysis Gamification Framework, the 8 core motivation drives are divided into white hat core drives (ie, epic meaning, accomplishment, and empowerment), which are designed to make users feel powerful, fulfilled, and satisfied. In contrast, black hat core drives (ie, scarcity, unpredictability, and avoidance) tend to make users feel obsessed, anxious, and addicted. The remaining 2 drives (ie, ownership and social influence) are neutral and do not fall under either category.

**Table 2.** Summary of the themes, subthemes, and detailed quotations in domain 2 (gamification design insights based on the Octalysis Framework).

Themes and subthemes	Supporting quotations
Overall attitudes	“I feel pretty good about it. It might be better than just using something basic, like providing video instructions alone.” (P2, male) “I think it's great because I really love playing games myself.” (P6, female) “For me, it’s because I value my time a lot. I wouldn’t want to spend too much time on this.” (P4, female) “I hope the game’s process and content can be simplified, as it’s primarily an exercise app.” (P12, male) “I’d prefer it to be simpler, something that can seamlessly integrate into our daily life.” (P14, male)
White hat core drives	
Epic meaning	
Attitude	“It would be nice if it worked like this, you know, like in a game where you're playing something like CS. For example, if I weigh 200 pounds and can only reach the first level, but after walking 10,000 steps today, I can move on to the second level. The second level could look more luxurious, with lots of rewards that I can claim.” (P3, male) “I’m not really a fan of these kinds of games, so I probably wouldn’t enjoy this one either. It doesn’t motivate me much, even if you added a storyline.” (P6, female)
Simplicity	“Exercise is already tiring enough, so why add all these flashy features? The simpler, the better. As you mentioned with the tree-growing idea, just seeing progress little by little can be enough. If it's too complicated, it just wastes time, and people won’t want to engage with it.” (P5, male)
Accomplishment	
Points	“From my perspective, I don't care about the extra points; what's important is that I want to exercise. Even now, I feel like exercising because I believe it benefits my body, so I just go ahead and do it. The number of points doesn't matter much.” (P9, female) “If it were possible to exchange points for physical rewards or other things, it would be more motivating for users to earn points.” (P12, male)
Progress bar	“I think that since it's my own goal, having a progress bar would make it clearer and more straightforward.” (P10, female) “The progress bar allows me to see my progress, but when I see that there's still a long way to go, I feel a sense of difficulty and pressure. However, with points, I don't feel this pressure.” (P8, male)
Leaderboard	“If there's a leaderboard with points, comparing myself to friends or even strangers can be motivating. When this kind of system is in place, I might feel a little pressure to rank higher, driven by competition or even a bit of vanity.” (P12, male) “But honestly, I think the leaderboard is too competitive. It just feels overwhelming, and I don’t like it.” (P6, female) “A real-time leaderboard can be particularly motivating, as you might be very close to second place and could potentially surpass them. At that point, you would likely choose to check in more often in order to overtake them.” (P7, male)
Badges	“A meaningful badge, especially one like the ones I got for running before, which are specific and limited, like a special badge for running during the Dragon Boat Festival. It's a bit like collecting.” (P7, male)
Reward system	“Something visually appealing, perhaps with a higher rarity, would be great. The simpler the reward, the better. If the reward system is too complicated, it just becomes confusing, and I might end up not wanting it at all.” (P2, male)
Empowerment	
Narrative setting	“The scenario setup should certainly allow for choice, right? Because there are 50 scenarios, and through effort, I can only reach 10 of them. The rest are unattainable, so I would just choose the 10 that I want to focus on.” (P3, male)
Goal setting	“Progressive goals are better, so that I don’t stop after just a day or two.” (P1, female)
Action planning	“It would be ideal if you could create a basic, foundational health plan or goal based on some basic information I provide. I would then be able to adjust it according to my own needs and preferences. Essentially, you would provide a framework, and I could make flexible adjustments within that structure. This approach would work better for me.” (P4, female) “What’s important in making a plan is how to connect it with daily habits.” (P7, male)
Black hat core drives	
Scarcity	
Closed group	“I don't accept it. It should be voluntary—you can join if you want. But if there are thresholds, it's like separating the top students from the bottom ones.” (P7, male)
Limited rewards	“There was a cycling challenge before that I tried. It started with a 10-kilometer challenge, then 20 kilometers, 50 kilometers, and different levels of difficulty. If you completed them, you could go to an offline store to claim your reward. A lot of people managed to stick with it for more than half, so the participation was pretty high. The rewards were limited, though, and many of them got snapped up quickly.” (P7, male)
Outdated points	“If I had the choice to set it myself, I would accept the rules. It would indirectly encourage me to exercise. If I don't exercise, I would lose the corresponding points, but if I do exercise, I would earn more points than I lost.” (P12, male)
Time-limited challenges	“In my view, for a challenge like this, is the reward given to anyone who meets the requirements, or is it just for a select few? Are the spots limited? I think a lot of people will be drawn in by the rewards. If the rewards aren’t attractive, though, I doubt many people would be willing to participate.” (P13, male) “But the time span needs to be long enough. For example, if you only give one or two days, and I happen to be on a business trip during that time, then I simply won’t be able to complete it.” (P8, male)
Unpredictability	
Random tasks	“There’s no penalty for failure—in other words, if you don’t complete it, no points will be deducted. That’s acceptable.” (P12, male)
Random rewards	“Rewards can be divided into two types, kind of like a blind box. There are hidden or special editions—if you want one of those, you can pay a higher price to get it. But if you’d rather pay less, then you rely purely on luck to draw one.” (P11, male)
Avoidance	
Points loss	“I don't want there to be any points that can be deducted in this process. After all, the points I earn are the result of my own effort.” (P6, female) “If you don't complete it, there could be a penalty or a supervision mechanism, which encourages you to finish the task. Of course, the points deducted shouldn't be too many—just a symbolic loss. However, that sense of loss might motivate you to push yourself to complete it.” (P8, male)
Ownership and social influence	
Ownership	
Exchangeable points	“It mainly depends on what you can use the points for. If you're collecting points to exchange for a specific item, you'll definitely work harder to earn those points in order to get that item.” (P13, male)
Avatar	“I would prefer it to be more customizable, like being able to change outfits. For example, you could earn things like different hairstyles or clothes through exercise milestones.” (P10, female) “You just input your data in, and that's me, even though it's virtual.” (P3, male) “It might just be that rarity is more important. Some things might be ugly, like certain luxury items, but because they're rare, people still really want them.” (P2, male)
Social influence	
Attitude	“This doesn’t really appeal to me. If it's someone I know, I wouldn't want them to know my step count or anything like that. And if it's a stranger, I have no desire to share or engage in any kind of exchange.” (P1, female) “I think mutual supervision is fine, and it's okay to check in with each other to complete tasks.” (P7, male) If it’s something like posting on WeChat, I think that would be better. After all, exercise is also a chance to connect and interact. A lot of people end up becoming friends through things like running, and I think that’s great.” (P8, male)
Mentorship	“Actually, it was a friend who encouraged me to start running. If I didn’t have anyone pushing me, I definitely wouldn’t have kept going. At the beginning, there are so many challenges, and it’s easy to give up. But my friend kept encouraging me and guiding me.” (P4, female)
Offline social activities	“Meeting some strangers can be quite exciting, because life can be too mundane at times. Sometimes, having something new and fresh can really make a difference.” (P4, female) “It's not limited to the types of activities we organize, such as sports. It could also include hosting lectures, either online or offline. For example, as mentioned earlier, a lecture about the relationship between exercise and hypertension could be held, either online or in person—both options are possible.” (P14, male)
Chat zones	“In a group of 50 people, everyone can have a conversation. For example, if someone is 50 years old and hasn’t seen much change after three months of exercise, and I’m 36, and after two months, I’ve seen good results, I could share my experience in just a few simple words.” (P3, male)

#### White Hat Core Drives (Epic Meaning, Accomplishment, and Empowerment)

Participants had mixed views on epic meaning gamification. While some found story-driven progress motivating, others preferred simpler designs, emphasizing that excessive storytelling could complicate the exercise experience and reduce engagement. Participants expressed diverse views on accomplishment-related gamification elements, such as points, progress bars, leaderboards, badges, and rewards. While some found these features motivating, others felt they could create pressure or distraction. They preferred meaningful, visually appealing, and simple rewards, such as limited-edition badges or tangible incentives, to enhance a sense of accomplishment. In addition, participants emphasized the need for empowerment through personalized, flexible goal setting and action planning. They preferred progressive goals, customizable plans that balance system guidance with personal choice, and seamless integration of exercise routines into daily life to cultivate exercise habit.

#### Black Hat Core Drives (Scarcity, Unpredictability, and Avoidance)

Participants showed mixed attitudes toward scarcity elements. They preferred voluntary participation over closed groups, viewed limited rewards as motivating when fairly attainable, and generally supported outdated points as a self-regulated incentive. For time-limited challenges, they emphasized the need for appealing rewards and flexible durations to fit diverse schedules. Participants favored random rewards without penalty, suggesting that unpredictability could enhance engagement if implemented positively. Creative ideas, such as blind box–style rewards, were viewed as appealing ways to maintain user interest and excitement. Moreover, participants expressed mixed views on avoidance mechanisms. While some opposed point deductions, others accepted small, symbolic penalties as a mild motivator to encourage task completion without causing frustration.

#### Ownership and Social Influence

Participants valued a sense of ownership through features such as exchangeable points and customizable avatars. They preferred avatars that reflect personal identity and include collectible or rare items, enhancing motivation and emotional connection to the app. Participants expressed diverse views on social influence in gamified exercise apps. While some preferred privacy and minimal sharing, others valued mutual support, peer interaction, and community features. Many favored social activities and opportunities for mentorship or experience sharing, viewing them as effective ways to enhance motivation and engagement.

### Domain 3: Psychological Needs Satisfaction in SDT

#### Overview

According to SDT, individuals engage in behaviors because their basic psychological needs (ie, autonomy, competence, and relatedness) are fulfilled by the external environment. We interviewed participants on these relevant aspects. Themes, subthemes, and detailed quotations in domain 3 were summarized in Table S2 in Multimedia Appendix 3.

Participants highlighted the importance of autonomy in exercise, preferring activities that align with their personal interests and preferences. They believed that such self-chosen exercises enhance enjoyment and support long-term engagement. Participants associated competence with mastering technical skills, gaining control over exercises, and experiencing a sense of ease. They also highlighted that perceived benefits, including improved sleep quality, overall health, emotional well-being, and weight management, further strengthened their motivation to continue exercising. Participants also emphasized that social connections, atmosphere, and group activities enhance motivation for exercise. Engaging with friends or workout partners made PA more enjoyable and supportive.

#### Extrinsic Motivation

Participants were primarily motivated by extrinsic factors such as weight management, physical health, and external support. Environmental convenience, personal trainers, and structured exercise programs helped maintain engagement, though many relied on external drives to stay active.

## Discussion

### Principal Findings

In this qualitative study, we interviewed 14 prehypertensive individuals, providing valuable insights into their needs and preferences for exercise apps, gamified design considerations based on the Octalysis Gamification Framework, and basic psychological needs satisfaction and motivation according to SDT.

To the best of our knowledge, this study is among the first to systematically integrate these 2 theoretical frameworks to understand user requirements for digital health interventions in prehypertension management. Our research advances both theoretical understanding and practical application by revealing how the Octalysis Gamification Framework and SDT can synergistically inform exercise behavior change interventions, while providing evidence-based guidance for app design. The design implications discussed below should be interpreted according to the study context, which involved a small qualitative sample of individuals with prehypertension recruited online in mainland China. Notably, these findings are most applicable to prehypertension populations in similar Chinese digital health contexts. They are not directly generalizable to offline-recruited prehypertension groups, individuals from other cultural backgrounds, or nonexercise-focused gamified apps.

### Interpretation of Findings

#### Objective 1: Exercise App Needs and Design Preferences Among Individuals With Prehypertension

This section addresses the first objective of the study, which aimed to explore the needs and design preferences for exercise apps among individuals with prehypertension.

Since the emergence and popularity of the concept of user-centered design, many qualitative studies have been conducted to explore individuals’ needs and preferences for app design, especially in the health sector, such as apps targeting PA [27-30]. According to previous findings from interviews conducted with individuals who have chronic obstructive pulmonary disease, physical disabilities, and frailty, similar results were observed, such as the need to motivate users to engage in exercise and the importance of incorporating gamification and social elements into these apps. These functional requirements were also identified in our interview results. However, different populations have varied demands depending on their primary exercise goals.

In our study, we focused on prehypertensive individuals, whose primary motivation for exercising was to lower BP and prevent the incidence of hypertension. As a result, they place high value on their exercise data and require feedback like weekly or monthly reports of their behavior, which differs from other populations. Moreover, physiological data such as BP, weight, and heart rate were particularly important for them to monitor their health status and the effectiveness of exercise. Therefore, they expressed a strong desire to integrate wearable devices into these apps. From the participants’ perspectives, they want the device to be designed to be portable and comfortable, so that it does not hinder their use, which is consistent with previous findings [31,32]. With regard to the functions of these wearable devices, they should be connected to apps to present exercise and physiological data, thereby informing users about their physical conditions. However, there are many challenges when incorporating wearable devices into apps, such as data privacy, standardization, accuracy, and seamless integration with existing systems, all of which should be carefully considered by software developers [33,34].

#### Objective 2: User-Centered Gamification Design Strategies Based on the Octalysis Framework

In line with the second objective, this section discusses user-centered gamification design strategies based on the Octalysis Framework.

According to previously published studies, most qualitative studies have mentioned the need for integrating gamification nudges or elements into apps, but they lack specific suggestions on how to design gamification techniques, leaving software developers confused about what to do next [32,35,36].

In our study, participants have not only provided their attitudes and suggestions about existing gamification functions and techniques, but also offered innovative ideas for the design of gamification rules, reflecting a participant-incorporated design perspective. For example, participants expressed that they want the gamification techniques used in the apps to be simple, easy to understand, and easy to start, which aligns with a previous systematic review stating that gamification systems should be easy to use and simplify content [37].

Almost all participants expressed very positive stances toward gamification used in these apps, consistent with previous studies [38,39]. In addition, varied attitudes were observed toward different gamification motivational drives, based on participants’ previous experiences, personal preferences, and personalities. With regard to specific motivational drives, participants expressed conservative attitudes toward narratives, as they think it is difficult to design an engaging storyline. However, a story without attraction would be ineffective, which aligns with previous studies that emphasize the effective use of narrative element [40].

Accomplishment is the most commonly used motivational drive in gamified apps, with traditional points, badges, and leaderboards elements underpinning this drive. During the interviews, most participants had encountered these elements in their previous experiences. They provided creative suggestions for enhancing the motivational functions of these techniques, such as exchangeable points, rare and meaningful badges, and dynamic leaderboards. These insights are valuable for gamification designers and software developers.

Avatars are a common gamification element in the ownership drive, and participants mentioned that they can be dynamic, personalized, and rare to attract users, a technique emphasized in a previous study [41]. Social influence is a core motivational drive in the Octalysis Gamification Framework, and social factors should always be considered when triggering a behavior. Based on our interview observations, the acceptance of social elements is highly related to a person’s personality, which is confirmed by previous studies [42]. Therefore, generating personality-matched social designs is worth exploring in the future.

In addition to other commonly used social features in apps, such as group tasks, friend circles, and chat zones, offline social activities and mentorship received very positive acceptance, which should be considered in future designs. Regarding black hat drives, participants did not show any notable preferences for these motivational drives, mainly because they are driven by the fear of losing. If these techniques are used appropriately, they may produce unexpected results.

#### Objective 3: Supporting Motivational Transformation Through Exercise Apps: The SDT Perspective

Addressing the third objective, this section elucidates how exercise apps may facilitate motivational transformation in individuals with prehypertension. Participants’ engagement in exercise was closely linked to the satisfaction of autonomy, competence, and relatedness.

With regard to autonomy, participants mainly valued a sense of volition, preferences, and interests, which shows similar results to a previous study that used SDT to explain trial retention behavior [43]. The key difference between exercise and retention behavior is the complexity of exercise and its various types. Therefore, the future designs should emphasize exercise choices based on users’ interests.

Participants also illustrated the importance of competence satisfaction. Many expressed that their progress or a sense of achievement in handling more difficult tasks would be a significant reason for them to continue exercising, which aligns with the results of a previous study [44]. Accordingly, progressively difficult tasks could be considered in future challenge designs. Relatedness, or the connection with others, should not be neglected when explaining behavior. Many participants shared that the involvement of others would be a notable drive to promote their exercise, a result also confirmed by quantitative data [45]. Incorporating social factors appropriately to satisfy the needs of different groups warrants consideration.

According to our interview results, most participants exercise due to extrinsic motivations, such as weight management and health values, while intrinsic enjoyment was less commonly observed. However, intrinsic motivation is predicted to support long-term, habitual physical activity [46].

Therefore, future research should focus on strategies that can transform extrinsic motivation to intrinsic motivation. With the rapid popularity of digital technologies, integrating these technologies with behavior change strategies could play a crucial role in influencing behavior and motivating participants. Digital tools, such as apps, large language models, and wearable devices, combined with strategies such as gamification, nudge, and behavioral economics, could contribute to addressing physical inactivity by fostering participants’ intrinsic motivation.

### Strengths and Limitations

The qualitative study has several strengths. In the digital age and the era of technological explosion, integrating user-centered design principles into technology development is crucial. The study used a hybrid inductive or deductive thematic analysis to explore prehypertensive individuals’ needs and preferences for gamified exercise apps through semistructured interviews. Based on SDT and the Octalysis Gamification Framework, the study enriches the theoretical basis for behavior change experts and provides practical design insights for software developers. It is timely and aligns well with global trends.

The study also has limitations. We recruited participants through an online poster, and individuals who responded were more likely to pay attention to their physical health compared with the general populations. Therefore, the insights derived from the included participants may not be generalizable to the broader population. Intervention designs based on these insights may not always be effective. Future quantitative studies, including participants from diverse cultural backgrounds and regions, should be conducted to validate our findings.

Moreover, since motivation and psychological need satisfaction are likely to change over time, longitudinal studies could provide a better understanding of exercise behavior according to SDT. Additionally, ethical considerations warrant reflection when designing gamified exercise apps, as some participants expressed concerns about pressure, competition, and the possibility that excessive entertainment-oriented gamification may weaken the app’s exercise-focused purpose. Future researchers remain attentive to the potential psychological burden of such apps and ensure that participation is voluntary and supportive. A further limitation is that interrater reliability (eg, Cohen κ) was not formally calculated. Although discrepancies in coding were resolved through consensus among researchers, future studies can incorporate statistical measures of interrater reliability to strengthen methodological rigor.

### Conclusions

This theory-informed qualitative study is among the first to explore the needs and preferences of individuals with prehypertension for gamified exercise apps by integrating the Octalysis Gamification Framework with SDT. The findings suggest that successful gamified exercise apps for prehypertensive individuals may benefit from going beyond traditional game mechanics to address deeper psychological needs. Participants emphasized the importance of personalized exercise programming, robust health monitoring capabilities, and human-centered gamification designs that support autonomy, competence, and relatedness. Satisfaction of these basic psychological needs is critical for transforming extrinsic motivations, such as weight management and health values, into intrinsic enjoyment of exercise. By integrating a theoretical basis with user-centered perspectives, this study provides context-specific design implications for future development of gamified exercise apps tailored to prehypertensive individuals in similar digital health contexts.

## Supplementary material

10.2196/86793 Multimedia Appendix 1 Interview guidelines.

10.2196/86793 Multimedia Appendix 2 Themes of domain 1.

10.2196/86793 Multimedia Appendix 3 Themes of domain 3.

10.2196/86793 Checklist 1 COREQ checklist.

## Abbreviations

COREQ: Consolidated Criteria for Reporting Qualitative Research
JARS-Qual: Journal Article Reporting Standards for Qualitative Research
PA: physical activity
SDT: Self-Determination Theory
WHO: World Health Organization
