# Supplementary material for: Design Requirements for Gamified Exercise Apps for Adults With Prehypertension Based on the Octalysis Framework and Self-Determination Theory: Qualitative Interview Study
Source: JMIR Serious Games. 2026 Feb 25;14:e86793. doi: 10.2196/86793 (PMC12935292; doi:10.2196/86793)
Supplement: Multimedia Appendix 1 [file games-v14-e86793-s001.docx]

**Interview guidelines**

**Basic Questions (Introduction)**

1. How many times a week do you usually exercise? What type of exercise do you do? How long dose each session last? (Exercise habits)
2. Do you think exercise is important to you? Why? (Perception of exercise)

- If you don’t exercise regularly, what are the main reasons?
- If you do exercise regularly, what are your main reasons?

**Part One: Exercise Behavior**

1. Autonomy

- Do you have a favorite type of exercise? What is it, and how did you discover that you enjoy it?
- What kind of exercise are you currently doing? Was this your own decision?
- Have you been influenced by recommendations from doctors, family, or friends?
- Do you prefer to plan your exercise by yourself, or would you rather receive suggestions or guidance — for example, from family, friends, or application recommendations?

1. Competence

- How do you feel before and after exercising? Have you noticed any changes in your body, mood, or energy levels?
- During your exercise routine, do you feel that you’re making progress? How do you know that you’re improving?

1. Relatedness

- Have you ever exercised with others? Do you think exercising with others is important to you? Why or why not?
- Is there anything related to exercise that I haven’t asked but you’d like to share?

**Part Two: Needs and Preferences for Exercise Applications**

1. Have you ever used any exercise-related apps or mini-programs? What was your experience like? (Past experience)

- If not, would you be willing to give them a try?
- If yes, what motivated you to use them? Which specific features did you use?

1. Before using an exercise app, what kind of support or advice would you expect it to provide? (Content needs)

- For example, exercise-related knowledge?

1. What features do you think an exercise app should have? (Functional needs)

- For example, planning exercise, tracking exercise data, or motivational functions?

1. What kind of app interface do you prefer? Including font style, colors, icons, etc. (Design preferences)

- Which is more important to you: appearance or ease of use?

1. What goals would you like to achieve through an exercise app? Why?

Is there anything else you’d like to add about your needs and preferences regarding exercise app design?

**Part Three: Gamified Incentive Mechanisms**

Do you play games regularly? What games have you played that made you feel impressive? How would you feel if an exercise app included gamified elements? (Gamification acceptance)

Gamification Factors

1. Meaningfulness: How would you feel if the exercise app included a storyline? Would you like this design?
2. Sense of Achievement: How do you feel about having features like points, leaderboards, badges, or progress bars in the app?

- Another aspect is rewards—what type of rewards would motivate you? Do you prefer virtual rewards, such as badges or titles, or real-world rewards?

1. Empowerment: Would you like to receive exercise feedback from a fitness tracker while using the app?

- Do you prefer setting your own exercise goals, or would you rather the app assess you first and then set goals for you, or allow you to choose your own goals?

1. Ownership: What do you think about having virtual avatars or items in the app?

- What would your ideal virtual avatar or item look like? Would it be a simple figure like a stick figure, an avatar, or a digital human?

1. Social Influence: Would you like the app to have social features? Would you like to challenge with friends together?

- How do you feel about social features in the app, such as checking in with friends, liking and commenting, team challenges, or exchanging virtual social gifts?

1. Scarcity: Do you like time-limited challenges or tasks with expiration dates for exercise points, for example, limiting specific types of exercise to a certain number of days?
2. Unpredictability: Would you like the app to include random rewards or tasks?

- For example, would you enjoy not knowing the rewards after completing a task or the prizes available for redeeming points?

1. Avoidance: What do you think about incorporating money or points deductions in the app?

- For example, losing points or money for not completing tasks—would this design motivate you, or would it create more pressure?

Do you have any overall preferences regarding the gamified settings, such as a desire for simplicity? Is there anything else you’d like to add about the gamified settings?
