# Supplementary material for: Design Requirements for Gamified Exercise Apps for Adults With Prehypertension Based on the Octalysis Framework and Self-Determination Theory: Qualitative Interview Study
Source: JMIR Serious Games. 2026 Feb 25;14:e86793. doi: 10.2196/86793 (PMC12935292; doi:10.2196/86793)
Supplement: Multimedia Appendix 2 [file games-v14-e86793-s002.docx]

| **Table S1.** Summary of the Themes, Subthemes, and Detailed Quotations in Domain 1 (Needs and Preferences for Exercise Apps). | |
| --- | --- |
| Themes and subthemes | Supporting quotations |
| Need for exercise application  **Exercise guidance and behavioral support** | |
| Real-time exercise posture feedback | *“Functionally speaking, there are certain expectations I have. For instance, when I’m exercising, I hope the app can guide me on which movements are standardized”.* (P1, female) |
| Calendars for automatic reminders | *“I’d like it to integrate with the calendar feature, since most calendars already have reminder functions. Given that I’ve granted permission, it could send me daily reminders or set a small alarm in my schedule”.* (P10, female) |
| Individualized exercise plans | *“It would be helpful if it could assist in planning by inputting my details, such as my age, and tailor a plan for me. For example, it could outline a weekly, monthly, or even yearly plan and provide an estimate of the expected results over time”.* (P3, male) |
| **Exericse data analytics and physiological monitoring** | *“After exercising, it would calculate the calories burned”.* (P9, female) |
|  | *“For example, if I go for a jog, I would activate the outdoor running mode and record the distance of my jog, as well as track the data from each session”.* (P13, male) |
|  | *“The purpose of recording this data daily is to create a weekly and monthly report, which shows my progress over time”.* (P7, male) |
|  | *“I would suggest incorporating changes in certain indicators. For instance, after running for a while, if my blood pressure drops from 140 to 130, it would be helpful to track this. Unlike other apps that only show my exercise trajectory and habits, having data on how my health indicators are changing could serve as a positive motivator for my future workouts”.* (P14, male) |
| **Health information aquisition** | *“I hope you can integrate some of the previous research findings, including both successful and unsuccessful studies, as well as fundamental knowledge about hypertension, to provide a comprehensive overview. It would be helpful to know which target groups can benefit from this”.* (P3, male) |
| User experience optimization | *“It should allow me to quickly and comprehensively understand its features, making it easier to learn and use. Currently, there are still many aspects that I am not fully clear about”.* (P4, female) |
|  | *“What matters to us more is the practicality of the app, not its appearance. If too much focus is placed on the design, you might be prioritizing its visual appeal over the core content and functions that you want to showcase”.* (P9, female) |
|  | *“I hope the app doesn’t have too many features but rather tailors to different user groups with specific options, and I would also like the app to have stronger interactivity”.* (P1, female) |
| Design Preferences for Exercise App | *“I have a small suggestion regarding your interface design. It shouldn’t just be a standard table like everyone else’s, as that can feel distant. You need something more humanized and humorous—perhaps some attractive illustrations and encouraging messages”.* (P11, male) |
|  | *“I think the font should be simpler”.* (P6, female) |
|  | *“As for the icons, it could be something like a person running. For example, when you open the app, it could show a person running, which I think works well. Since the main purpose of the app is to promote exercise, and running or walking are the simplest forms of exercise, it would be great to show a person running, as it’s something anyone can do right away”.* (P3, male) |
|  | *“I think green would be the most fitting, as green is soothing to the mind and represents health, which aligns with the idea of wellness being connected to nature”.* (P9, female) |
|  | *“If I had to choose, I’d go with colors like red or orange, as they look vibrant and energetic*.” (P6, female) |
|  | *“It would also be nice to integrate features like DeepSeek conversations. I think that would work well too”.* (P1, female) |
| Needs for Wearable Device | *“I actually feel quite good wearing it. A slightly thinner and smaller wristband would be ideal. It doesn’t need too many functions, just the ones we need”.* (P3, male) |
|  | *“I don’t wear the wristband at night anymore because it gets a bit too hot for me”.* (P8, male) |
|  | *“I mainly use it to track exercise-related metrics, such as heart rate, steps, distance, and other workout data”.* (P7, male) |
|  | *“When I wear the watch, I can clearly monitor my blood pressure and know when it’s not feeling right.”* (P4, female) |
|  | *“I think it’s really important to have alerts. For example, my watch has a feature that notifies me if my heart rate becomes too high. These reminders are necessary for me”.* (P8, male) |

Abbreviation: P, participant.
