# Supplementary material for: Design Requirements for Gamified Exercise Apps for Adults With Prehypertension Based on the Octalysis Framework and Self-Determination Theory: Qualitative Interview Study
Source: JMIR Serious Games. 2026 Feb 25;14:e86793. doi: 10.2196/86793 (PMC12935292; doi:10.2196/86793)
Supplement: Multimedia Appendix 3 [file games-v14-e86793-s003.docx]

| **Table S2.** Summary of the Themes, Subthemes, and Detailed Quotations in Domain 3 (Psychological Needs Satisfaction in SDT). | |
| --- | --- |
| Themes and subthemes | Supporting quotations |
| Psychological Needs Satisfaction in SDT |  |
| Autonomy  Sense of volition | *“Maybe I'm a more intuitive person; I just feel much better on my own and don’t really like being guided by others”.* (P7, male) |
| Preferences | *“Because I choose an exercise that suits me, running definitely doesn't work for me, so I opt for activities like hiking, walking, or cycling. I can still find an exercise that works for me”.* (P1, female) |
| Interests | *“I only learned how to play bowling after starting work, and I found it quite interesting. So, I gave it a try, and once I got the hang of it, I really enjoyed it. Over time, it became a hobby and a regular habit”.* (P4, female) |
| Competence  Technical challenges | *“I don’t feel like I’d choose running just to exercise. I’d definitely choose swimming instead, because I feel it’s more technical. So, when my skills improve, I get more excited, and that motivates me to keep going”.* (P6, female) |
| Gradual development of control | *“At the beginning, you might feel like you're not doing well, but after more practice, you'll start to figure out some tricks, like the angle, posture, direction, and strength. Over time, you’ll get better and develop your own methods. Overall, it’s really meaningful for your personal learning and growth”.* (P8, male) |
| The sense of ease | *“I tend to do a lot of easy exercises that don't require too much, and don't need a lot of energy or triggers to get started. The lower the energy required to start, the better”.* |
| Relatedness  Positive social atmosphere | *“I feel that making plans to go out with friends creates a more relaxed atmosphere, so hiking becomes more enjoyable in comparison”.* (P13, male) |
| Sense of group belonging | *“If you tell me to swim twice a week, I might resist, but if someone invites me to play volleyball and there's other people involved, I’d probably go”.* (P6, female) |
| Social connections | *“Having a buddy makes hiking difficult mountains more manageable. You can help each other out, offer emotional support, and encourage one another, so it doesn’t feel as monotonous”.* (P13, male) |
| Extrinsic motivation  External factors (e.g., weather) | *“I listen to music while running, and since the park is very close, it naturally creates favorable conditions, so I end up running”.* (P7, male) |
| Weight management | *“At that time, the main goal was to lose weight”*. (P14, male) |
| Improved physical condition | *“Doing yoga has helped me feel one benefit: my body isn't as stiff”.* (P4, female) |
| Health values | *“My body is in a bit of trouble — I not only have fatty liver, but my uric acid level is also very high, over 500. If I don’t lose weight soon, I might develop gout”.* (P11, male) |
| Supervised by a fitness coach | *“I used to sign up for personal training classes, and that was more of a passive approach — since the coach scheduled sessions with me, I ended up going regularly each week”.* (P7, male) |
| Exercise programs | *“When I follow an exercise program, my blood pressure goes down — but it depends on an external push. Once that external motivation is gone, I fall back into my old habits and stop exercising again”.* (P1, female) |
| Intrinsic motivation  Enjoyment | *“I’ve been running for three to five years now. Running was basically the first form of exercise I started with, and it makes me feel happy. It’s the kind of activity I genuinely want to do on my own initiative”.* (P4, female) |

Abbreviation: SDT, self-determination theory; P, participant.
